# Low-frequency ultrasound irradiation increases paclitaxel-induced sarcoma cells apoptosis and facilitates the transmembrane delivery of drugs

**DOI:** 10.3389/fphar.2022.1065289

**Published:** 2022-12-13

**Authors:** Tana Yang, Yixuan Zhang, Tan Wang, Mo Li, Ying Zhang, Dan Zhao, Libin Xu, Xiaobing Wang

**Affiliations:** ^1^ Beijing Children’s Hospital, Capital Medical University, National Center for Children’s Health, Beijing, China; ^2^ State Key Lab of Molecular Oncology, Laboratory of Cell and Molecular Biology, National Cancer Center/National Clinical Research Center for Cancer/Cancer Hospital, Chinese Academy of Medical Sciences and Peking Union Medical College, Beijing, China; ^3^ Department of Gynecological Oncology, National Cancer Center/National Clinical Research Center for Cancer/Cancer Hospital, Chinese Academy of Medical Sciences and Peking Union Medical College, Beijing, China; ^4^ Department of Orthopedic Surgery, National Cancer Center/National Clinical Research Center for Cancer/Cancer Hospital, Chinese Academy of Medical Sciences and Peking Union Medical College, Beijing, China

**Keywords:** low-frequency ultrasound irradiation, paclitaxel, sarcoma, apoptosis, drug delivery

## Abstract

Sarcoma is a malignant tumor derived from interstitial tissues and requires comprehensive treatment including chemotherapy. Paclitaxel (PTX) is an active agent against sarcoma, but its effect is not sufficiently acceptable and needs to be improved. Low-frequency ultrasound (LFU) has been documented to improve the efficacy of drugs by inducing reversible changes in membrane permeability; however, the effects of the combined use of LFU and PTX for sarcoma tumors remain unclear and warrant further investigation. We investigated the effects of 30 kHz LFU treatment combined with PTX on sarcoma cells A-204 and HT-1080 by analyzing *in vitro* apoptosis and cell growth inhibition rates, and determined their antitumor effects by examining tumor weights with or without LFU in the S180 sarcoma xenograft model. Drug concentrations in the subcutaneous tumors were measured using high performance liquid chromatography (HPLC). LFU combined with PTX significantly induced cell apoptosis, and blocked the cell cycle of sarcoma cells in G2/M phase, and furthermore, inhibited the activation of JAK2/STAT3 signaling pathway. Meanwhile, LFU combined with PTX inhibited the expression of PD-L1 *in vitro*, suggesting the potential of enhanced antitumor immunity by this treatment. LFU combined with PTX significantly inhibited the growth of S180 tumors transplanted subcutaneously in Institute of Cancer Research (ICR) mice, and its enhanced effect may be associated with increased local concentrations of PTX in tumor tissues *in vivo*, with no significant adverse subsequences on body weight observed. We conclude that the combination of LFU and PTX has synergistic antitumor effects and is a candidate for subcutaneous treatment of sarcoma by further increasing the intracellular concentration of PTX.

## Introduction

Sarcoma is a malignant tumor derived from interstitial tissue with unknown etiology, features a short disease course and the cause can vary between individuals. It occurs mostly in the skin and subcutaneous tissues, and easily invades the surrounding soft tissue and develops rapidly. Paclitaxel (PTX) is broadly used for sarcoma tumors as a valuable chemotherapeutic agent (Zhang et al., 2012). Patients with sarcoma may achieve acceptable curative effect that undergo chemotherapy alone, while for most sarcomas, the outcome is often unsatisfactory and also accompanying adverse effect, and therefore the comprehensive treatment of new chemotherapeutic regimens was needed and crucial to improving the survival rate of sarcoma patients.

Low-frequency ultrasound (LFU), a non-invasive procedure that has been documented to increase the efficiency of drug passage into cells by inducing reversible changes in membrane permeability, could allow local treatment of tumors and reduce side effects of drug treatment (Shen et al., 2014; Schoellhammer and Traverso, 2016). In addition, as ultrasound acts locally on the tumor, it can minimize damage to the surrounding normal tissues (Xia et al., 2012; Sadeghi-Naini et al., 2013). Recently, ultrasound has become an effective clinical technique for adjuvant targeted therapy. Previous studies have shown that the non-cavitating mechanical influence produced by ultrasound can improve the drug concentration in the local tumor mass and strengthen the intracellular drug uptake (Hu et al., 2016; Yang et al., 2016). However, the effects of the combined use of LFU and PTX for sarcoma tumors remain unclear and warrant further investigation. Furthermore, the use of immune checkpoint inhibitors in the treatment of tumors is becoming more and more crucial. It is still unknown if LFU has the ability to change the tumor immune microenvironment, thereby enhancing the likelihood of success in treating sarcoma with immune checkpoint inhibitors. Therefore, the intention of this research was to assess whether LFU combined with PTX could act as a means of comprehensive treatment, for synergistically increasing the anti-tumor action of the drug and providing experimental framework for clinical management of sarcoma tumor.

## Materials and methods

### Chemicals and animals

PTX was purchased from Beijing Union Pharmaceutical (Beijing, China). Dimethyl sulfoxide (DMSO) was purchased from Beijing North Chemical Fine Chemicals Co., Ltd. (Beijing, China). Male ICR mice of 6–8 weeks were purchased from China Food and Drug Inspection Institute. The mice were housed in cages at 22 ± 1°C temperature and 50%–60% humidity room under a 12 h light-dark cycle, in the laboratory animal room of National Cancer Center in Chinese Academy of Medical Sciences. Animal treatment and surgical procedures were performed in accordance with “Principal of Laboratory Animal Care” from National Institute of Health and were approved by the Institutional Animal Ethics Committee [SYXK (Beijing) 2014-0003].

### Cell culture

The S180 murine sarcoma cell line was obtained from the Cancer Institute of the Chinese Academy of Medical Sciences (Beijing, China), which was established in 1978 at institute of zoology, academia sinica. S180 was cultured in RPMI-1640 with 25 mM HEPES and 2 mM L-glutamine (BIOROC, China). The human fibrosarcoma cell line HT-1080 and human rhabdomyosarcoma cell line A-204 were purchased from Cell Resource Center, IBMS, CAMS/PBMC. HT-1080 was cultured in MEM-EBSS with 1% Non-Essential Amino Acids. A-204 was cultured in McCoy’s 5A Media (Modified with Tricine). All media contained 10% fetal bovine serum and penicillin (100 U/ml)/streptomycin (0.1 mg/ml). All cells were cultured at 37°C in a humidified atmosphere containing 5% CO_2_.

### Western blots

The extraction of total cellular protein was processed by lysis buffer (1 mM Tris-HCL, pH 6.8, 10% SDS and 80% glycerin). BCA kit was used for the determination of protein concentration following the manufacturer’s instructions. Briefly, 10% SDS-PAGE gel was applied for separating the 30 μg total protein, and then proteins were electrophoretically transferred to PVDF membranes (Millipore; Burlington, MA, United States). In the subsequent step, the membranes were washed three times with TBST and blocked thoroughly with 5% skim milk, and then incubated with antibodies specific for Bcl-2, Bax, JAK, p-JAK, p-STAT3, c-MYC, PD-L1, GAPDH (Cell Signaling Technology, United States; 1:1,000 dilution) at 4°C overnight. Secondary antibodies were applied for 2 h after primary antibody incubation. The detection of protein bands was verified with enhanced chemiluminescence kit (ECL; ThermoFisher Scientific, Waltham, MA, United States).

### Cell apoptosis and cell cycle analysis

Each well of 6-well plates was seeded with A-204 and HT-1080 cells and cultured for 24 h. The growth period cells were added to wells of a 96-well plate, with 5 × 10^3^ cells dispensed into each well. Cell samples were randomly allocated to four groups with three parallel samples per group (Group I, control group containing untreated A-204 and HT-1080 tumor cells; Group II, A-204 and HT-1080 tumor cells treated with PTX; Group III, A-204 and HT-1080 tumor cells treated with LFU, 100 mW, 5 min; Group IV, A-204 and HT-1080 tumor cells treated with LFU, 100 mW, 5 min, Plus PTX). The apoptosis of cells was detected by Annexin V-FITC/propidium iodide (PI) double staining (Beyotime, China), and analyzed by flow cytometry. In parallel, we performed cell cycle assays on each group of cells, with A-204 and HT-1080 cells washed with cold PBS after collection and fixed overnight at 4°C in 70% ethanol. Then, cells were centrifuged at 1,000 g for 3 min, washed with PBS three times and then stained with Propidium for 15 min protected from light for flow cytometric detection.

### Cell viability assay *in vitro*

The A-204 and HT-1080 cells were seeded and operated as above, then cells were cultured in the 5% CO_2_ atmosphere at 37°C for 24 h in an incubator. CCK8 (10 μl) was added to 100 μl of medium per well and incubated with the cells for 1 h at 37°C and 5% CO_2_. The absorbance at 450 nm was detected using a microplate reader (Bio-Rad Laboratories; Hercules, CA, United States). The data were analyzed with GraphPad Prism software version 6.00 for Windows (La Jolla, CA, United States). The inhibitory rate (IR, %) was calculated as [(control group OD-experimental group OD)/(control group OD-blank group OD)] × 100. Combined treatment index (Q) = Eab/[(Ea + Eb)–Ea × Eb], where Ea is the IR of tumor cells treated with LFU, Eb is the IR of tumor cells treated with PTX, and Eab is the IR of tumor cells treated with LFU plus PTX. A Q value of 0.85–1.15 indicated additive effect of the combination therapy, Q value more than 1.15 indicated an enhanced effect, and Q value less than 0.85 indicated antagonism.

### RNA extraction and quantitative real-time PCR

In order to extract the total RNA, we used TRIzol (ThermoFisher Scientific, Waltham, MA, United States), and mRNA was reverse transcripted into cDNA applying the PrimeScript RT Reagent Kit (TaKaRa; Tokyo, Japan). Then Quantitative real-time PCR (qPCR) was run with the SYBR^®^ Premix Ex Taq™ (TaKaRa) on ABI QuantStudio5 (ABI; Indianapolis, IN, United States). Sequences of qPCR primers were presented as below: PD-L1- F (TGG​CAT​TTG​CTG​AAC​GCA​TTT); PDL1-R (TGC​AGC​CAG​GTC​TAA​TTG​TTT​T); GAPDH-F (GGA​GCG​AGA​TCC​CTC​CAA​AAT); GAPDH-R (GGC​TGT​TGT​CAT​ACT​TCT​CAT​GG). The threshold cycle (Ct) values for each gene were normalized to those of GAPDH, and the 2^−ΔΔCT^ method was used for quantitative analysis. The data were analyzed with GraphPad Prism software version 6.00 for Windows (La Jolla, CA, United States).

### Immuno-infiltration analysis

RNAseq data in level 3 HTSeq-FPKM format were obtained from TCGA (https://portal.gdc.cancer.gov/) SARC (sarcoma) project. RNAseq data in FPKM (fregments per kilobase per million) format were converted to TPM (transcripts per million reads) format with log2 (TPM + 1) transformation and filtering to remove control/normal data from subsequent studies. The data were analyzed using the GSVA package (v.1.34.0) in R software (v.3.6.3) and using the ssGSEA immuno-infiltration algorithm. Spearman’s correlation coefficient was used for correlation analysis and the correlation coefficient (r) was calculated.

### Animal grouping and experiment design for S180 sarcoma *in vivo*

Six–eight weeks Institute of Cancer Research (ICR) male mice were subcutaneously inoculated in the backside region with 5 × 10^6^ S180 sarcoma cells suspended in 0.1 ml PBS. The mice were randomly divided into four groups with eight mice each, including control group (C), LFU group (30 kHz, 0.75 W/cm^2^ intensity, and 15 min, every 3 days, q3d × 3), tumor circumference hypodermic injection of upper part (TCIUP) group (20 mg/kg PTX at 3-day intervals for a total of three doses) and TCIUP plus LFU group. Each mouse was anesthetized by intraperitoneal injection of 0.15 ml of 2% pentobarbital sodium. Next, we placed a therapeutic low-frequency transducer of approximately 15 mm diameter on the skin of the mice and covered the entire tumor load of each mouse with contact medicinal foam. When tumors started to develop, the diameters of the transplanted tumors were measured with electronic calipers every 3 days, and tumors volume were calculated as [1/2 (a × b^2^)], in which “a” is the longest diameter and “b” is the shortest diameter of the tumor. Then, mice were sacrificed at the end of the experiment (day 21) and the tumors were removed and fixed using 10% neutral buffered formalin for at least 24 h. The tissue samples were embedded in paraffin and sliced into 5 mm-thick sections, followed by H&E staining. Histopathological changes in all sections were observed and evaluated using a light microscope.

### High performance liquid chromatography and measurement of local drug uptake in tumor tissue

Drug loading assays were performed using an HPLC obtained from Waters Corporation (Waltham, United States). Chromatography was performed using a Model 515 HPLC pump, a Model 2,487 dual absorption detector, a Model 717 Plus autosampler, and a Kromasil-C18^®^ column (China Analytical Instruments Co., Ltd.). The mobile phase was acetonitrile: water (5:5) with a detection wavelength of 230 nm, a flow rate of 1.0 ml/min and a column temperature of 25°C. The PTX standard peak was 60.0 g/ml of PTX. Twenty microliters of each dilution were injected into the liquid chromatograph and the chromatograms were recorded. One section (*n* = 8) of each experimental tumor containing PTX was placed in a pestle and mortar, crushed and mixed with water (2 µl/mg of tumor tissue). After initial grinding, 2 µl of distilled water was added and grinding continued until the tumor material was thoroughly ground. The material was stirred, filtered and the supernatant injected into the column for analysis.

### Statistical analysis

Data are shown as mean ± standard deviation (SD). Student’s *t*-test and ANOVA were used to examine the differences between the treatment and control groups. Differences were considered significant only when the *p*-value < 0.05. The data were analyzed with GraphPad Prism software version 6.00 for Windows (La Jolla, CA). *p < 0.05, **p < 0.01, ***p < 0.001, ns not significant.

## Results

### Low-frequency ultrasound promotes paclitaxel-induced cell growth inhibition in sarcoma cell lines

**FIGURE 1:**
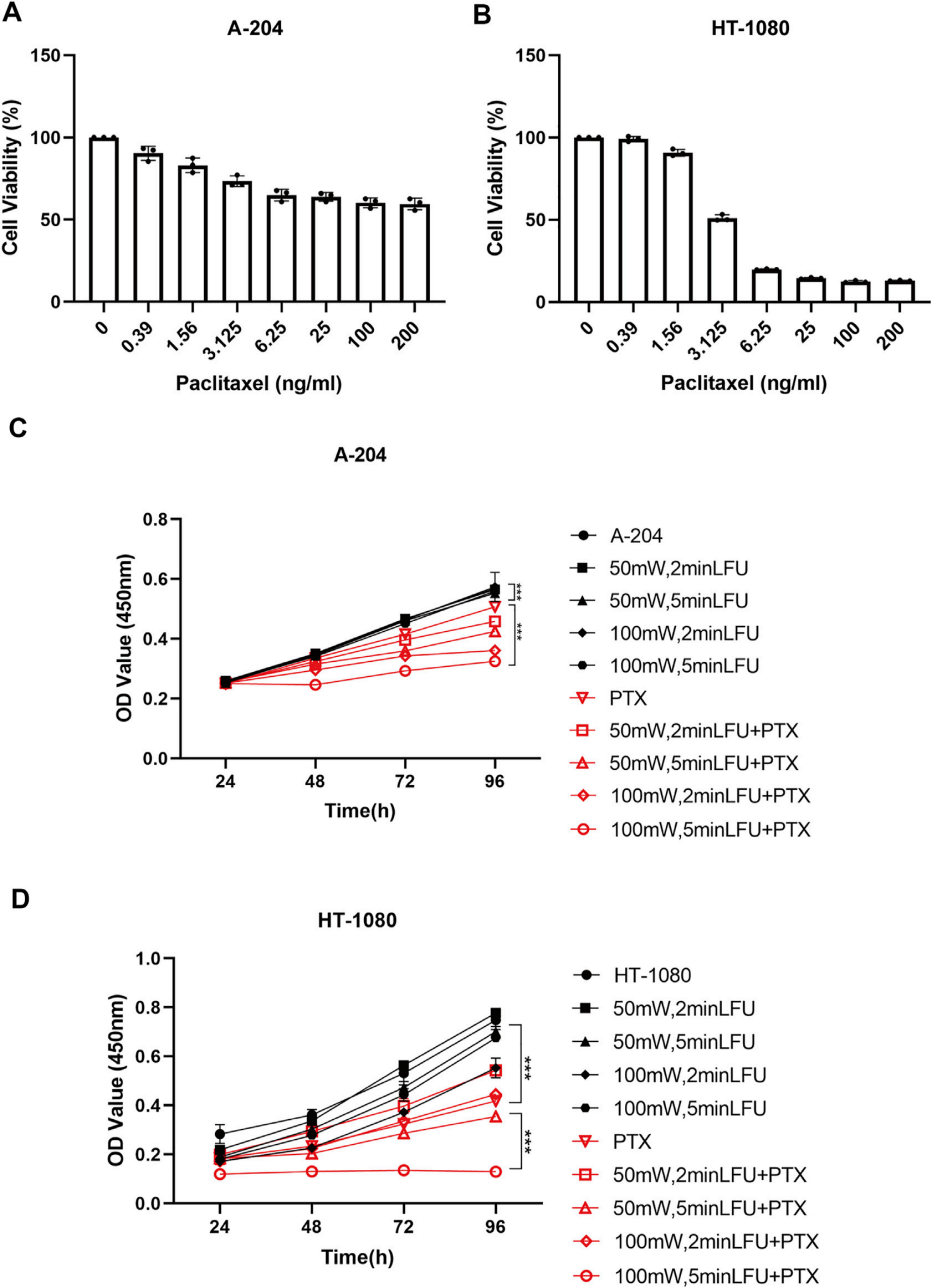
Inhibition by PTX combined with LFU on sarcoma cells. **(A)** Cell viability of A-204 cells treated with PTX. **(B)** Cell viability of HT-1080 cells treated with PTX. **(C)** Growth curves generated with OD450 data with the CCK8 assay for A-204 cells treated with LFU and PTX. **(D)** Growth curves generated with OD450 data with the CCK8 assay for HT-1080 cells treated with LFU and PTX. Data are shown as the mean ± SD. *p < 0.05, **p < 0.01, ***p < 0.001, ns not significant.

The sensitivity of A-204 and HT-1080 cell lines to different concentrations of PTX is shown in Figures 1A,B. We selected PTX concentrations of 800 ng/ml and 1 ng/ml as the subsequent working concentrations in A-204 and HT-1080 sarcoma cells, respectively. To determine the combination effects, sarcoma cells were treated with LFU alone, PTX alone and the combination of LFU and PTX. CCK8 assays demonstrated that LFU alone did not effectively suppressed the proliferation of sarcoma cell line for all groups (*p* < 0.05). In A-204 cells, the inhibitory rate of LFU 50 mW applied for 2 and 5 min was 5.08% ± 0.26% and 7.10% ± 0.69%, respectively (Table 1). The inhibitory rate of LFU at 100 mW was higher, increasing to 9.27% ± 0.49% and 10.3% ± 1.07%, respectively. The inhibitory rate of the 800 ng/ml PTX alone group was 53.7% ± 0.14%. In HT-1080 cells, the inhibitory rate of LFU 50 mW applied for 2 and 5 min was 2.67% ± 0.49% and 9.59% ± 0.55%, respectively. The inhibitory rate of LFU at 100 mW was higher, increasing to 11.9% ± 0.68% and 14.5% ± 0.17% respectively (Table 2). The inhibitory rate of the 1 ng/ml PTX alone group was 17.3% ± 0.61%. To further investigate whether LFU could enhance the tumor suppressive effect of PTX, we treated the cells with LFU in combination with PTX. The results revealed that LFU enhanced the inhibitory effect of PTX on proliferation and was directly proportional to the treatment dose, which was listed in the Tables 1, 2. The Q-value always exceeded 0.85 in the groups involving LFU plus PTX, suggesting a synergistic effect of LFU plus PTX and the combined anti-tumor effect was enhanced *in vitro*. Besides, we examined the proliferation for four consecutive days with A-204 and HT-1080, the results showed that LFU alone could not inhibit the proliferation significantly but LFU was able to enhance the inhibitory effect of PTX on proliferation (Figures 1C,D), suggesting a synergistic effect of LFU plus PTX.

**TABLE 1:** Inhibitory rate of PTX combined with LFU on A-204 cells *in vitro*.

Groups	A-204 cell inhibitive rate % ( $\bar{x}$ ± s)		
	LFU intensity	2 min	5 min
LFU	50 mW	5.08 ± 0.26	7.10 ± 0.69
	100 mW	9.27 ± 0.49	10.3 ± 1.07
PTX (800 ng/ml)		53.7 ± 0.14	
PTX (800 ng/ml) + LFU	50 mW	56.1 ± 0.90	59.8 ± 0.97
	100 mW	79.5 ± 0.23	80.6 ± 0.15
Q value (combined treatment index)	50 mW	1.00 ± 0.01	1.05 ± 0.02
	100 mW	1.37 ± 0.001	1.38 ± 0.02

**TABLE 2:** Inhibitory rate of PTX combined with LFU on HT-1080 cells *in vitro*.

Groups	HT-1080 cell inhibitive rate % ( $\bar{x}$ ± s)		
	LFU intensity	2 min	5 min
LFU	50 mW	2.67 ± 0.49	9.59 ± 0.55
	100 mW	11.9 ± 0.68	14.5 ± 0.17
PTX (1 ng/ml)		17.3 ± 0.61	
PTX (1 ng/ml) + LFU	50 mW	19.9 ± 0.99	28.1 ± 0.97
	100 mW	40.8 ± 0.70	92.4 ± 0.06
Q value (combined treatment index)	50 mW	0.95 ± 0.16	1.11 ± 0.06
	100 mW	1.50 ± 0.05	3.15 ± 0.05

### Low-frequency ultrasound increases cell apoptosis induced by paclitaxel in sarcoma cells

**FIGURE 2:**
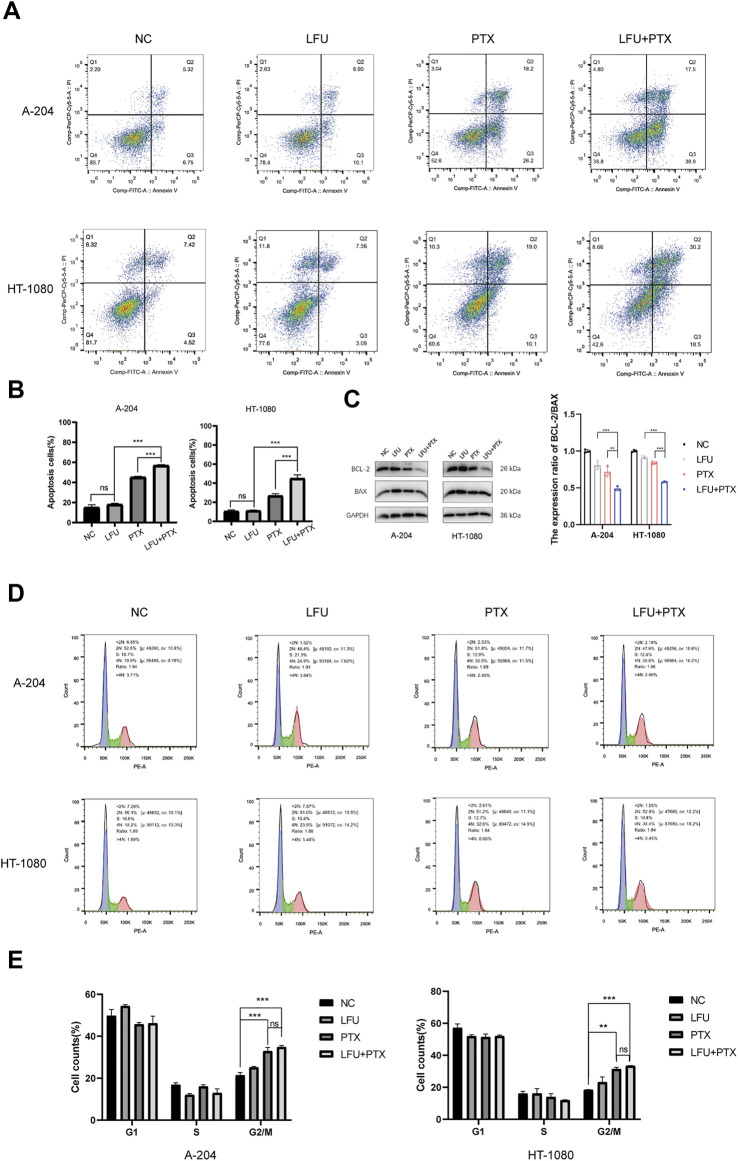
Effect of treatment with LFU + PTX on apoptosis of A-204/HT-1080 cells. **(A)** A-204/HT-1080 cells were treated with LFU, PTX or LFU + PTX for 48 h, stained with Annexin V-FITC and PI, and examined using flow cytometry. **(B)** Quantification of apoptotic rates in the experiments. **(C)** Expression levels of BCL-2 and BAX following treatment with LFU, PTX, and LFU + PTX. **(D)** Cell cycle distribution in A-204 and HT-1080 with LFU, PTX and LFU + PTX for 48 h. **(E)** The proportion of cell counts in different phases of cell cycle in A-204 and HT-1080 following treatment with LFU, PTX, and LFU + PTX. Data are shown as the mean ± SD. *p < 0.05, **p < 0.01, ***p < 0.001, ns not significant.

We next investigated how LFU affected the cell viability of sarcoma cells. Flow cytometry analysis showed that LFU alone could not increase the rate of apoptosis, but the combination group of LFU and PTX significantly increased the rate of apoptosis (Figures 2A,B), and Western blot analysis showed that the ratio of Bcl-2/Bax was significantly decreased in the combination group, indicating that LFU can synergize with PTX to promote PTX-induced apoptosis (Figure 2C). In addition, we performed A-204 and HT-1080 cell cycle assays after treatment with LFU alone, PTX alone, or in combination with LFU. The results showed that LFU alone could not block sarcoma cells in G2/M phase, but PTX alone and combined treatment significantly blocked sarcoma cells in G2/M phase (Figures 2D,E), LFU further enhanced the cell cycle arrest of PTX, while no statistical difference was shown.

### The combination of low-frequency ultrasound and paclitaxel inhibits the activation of JAK2/STAT3 signaling pathway

**FIGURE 3:**
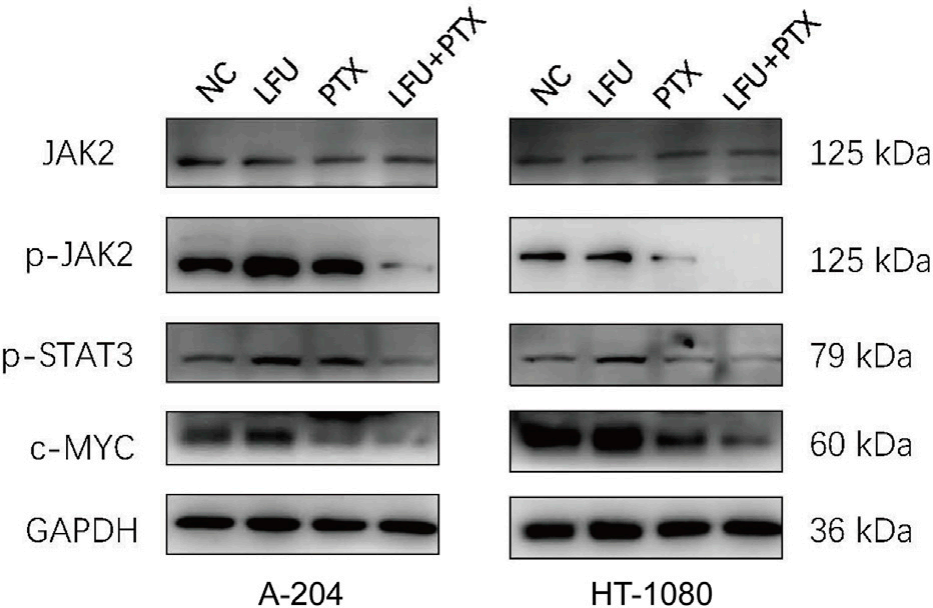
Western blot to detect protein expression of JAK2, p-JAK2, p-STAT3, c-MYC in A-204 and HT-1080 cells treated with LFU, PTX, and LFU + PTX for 48 h.

To address the mechanism of synergistic cell growth inhibition and enhanced apoptosis after treatment with LFU and PTX, sarcoma cells treated with either treatment alone or combination for 48 h were followed by assessment of JAK2/STAT3 signal pathway using Western blot analysis. The Janus kinases (JAKs) are major activators of signal transducer and activator of transcription (STAT) proteins. JAK2–STAT3 signalling is crucial for cancer development in both tumor cells and the tumor microenvironment, and both JAK and STAT3 have emerged as important targets for cancer treatment (Yu et al., 2014). We found that the expression of JAK2 was not changed but the p-JAK2, p-STAT3 was decreased significantly in the combination treatment (Figure 3), indicating the inactivation of JAK2/STAT3 pathway. *MYC* and *BCL-2* were downstream genes of JAK2/STAT3 signal pathway (Ok et al., 2014), and the Western blot analysis showed that the expressions of c-MYC and BCL-2 were significantly downregulated, which could enhance the apoptosis consequently.

### Low-frequency ultrasound combined with paclitaxel potentially promotes the anti-tumor immune responses

**FIGURE 4:**
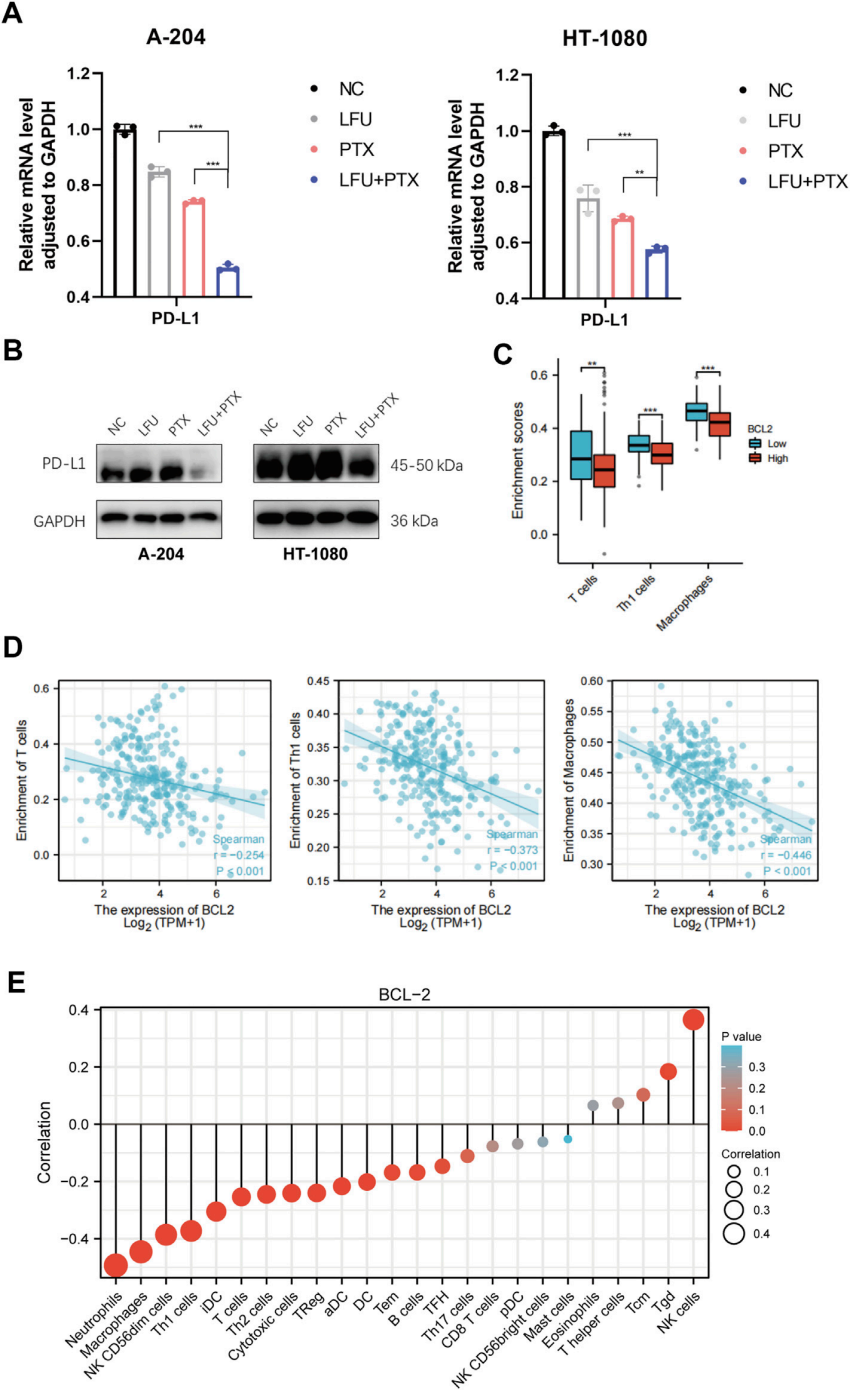
LFU combined with PTX promoted the anti-tumor immune responses **(A)** PD-L1 expression in A-204 and HT-1080 cells treated with LFU, PTX, and LFU + PTX for 48 h by using qPCR. **(B)** Western blot to detect protein expression of PD-L1 in A-204 and HT-1080 cells treated with LFU, PTX, and LFU + PTX for 48 h. **(C)** BCL2 expression versus immune infiltration of T cells, Th1 cells and macrophages. **(D)** BCL2 expression correlated with immune infiltration of T cells, Th1 cells and macrophages were calculated using the Spearman correlation algorithm. **(E)** The correlation of BCL2 expression with immune infiltration of 24 immune cells. *p < 0.05, **p < 0.01, ***p < 0.001, ns not significant. aDC, activated DC; B cells; CD8 T cells; Cytotoxic cells; DC; Eosinophils; iDC, immature DC; Macrophages; Mast cells; Neutrophils; NK CD56bright cells; NK CD56dim cells; NK cells; pDC, Plasmacytoid DC; T cells; T helper cells; Tcm, T central memory; Tem, T effector memory; Tfh, T follicular helper; Tgd, T gamma delta; Th1 cells; Th17 cells; Th2 cells; Treg.

Studies have shown that STAT3 has a crucial role in stromal cells, including immune cells, which are recruited to the tumor microenvironment to promote tumor progression (Kortylewski and Yu, 2008; Herrmann et al., 2010). Importantly, STAT3 activation also functions as a potent immune checkpoint for multiple antitumor immune responses (Kortylewski et al., 2005; Yu et al., 2009). In this study, we found that combined treatment with LFU and PTX inhibited the activation of JAK2/STAT3 signaling pathway and decreased the expression of downstream genes *MYC* and *BCL-2*. Previously, MYC was shown to regulate the expression of an immune checkpoint protein on the surface of tumor cells, the adaptive immune checkpoint PD-L1 (programmed death ligand 1). MYC inactivation in mouse tumors downregulated PD-L1 expression and enhanced the anti-tumor immune response (Casey et al., 2016). Therefore, we next explored the correlation between target genes, c-MYC and BCL-2, and antitumor immune responses. The results indicated that PTX inhibited the expression of PD-L1 and LFU could enhance the effect of PTX, at both mRNA and protein level (Figures 4A,B). Next, we explored the correlation between BCL-2 and immune cells infiltration since *BCL-2* is downstream target gene of JAK2/STAT3 signaling pathway. The results showed that the expression of BCL-2 in tumor cells was negatively correlated with the presence of T cells, Th1 cells and macrophages (Figures 4C,D), and positively correlated with T helper cells and NK cells (Figure 4E), which indicated the promotion of anti-tumor immune responses with the combination treatment of LFU and PTX.

### Low-frequency ultrasound combined with paclitaxel inhibits S180 tumor growth *in vivo* and enhances the local paclitaxel concentrations in tumor tissues

**FIGURE 5:**
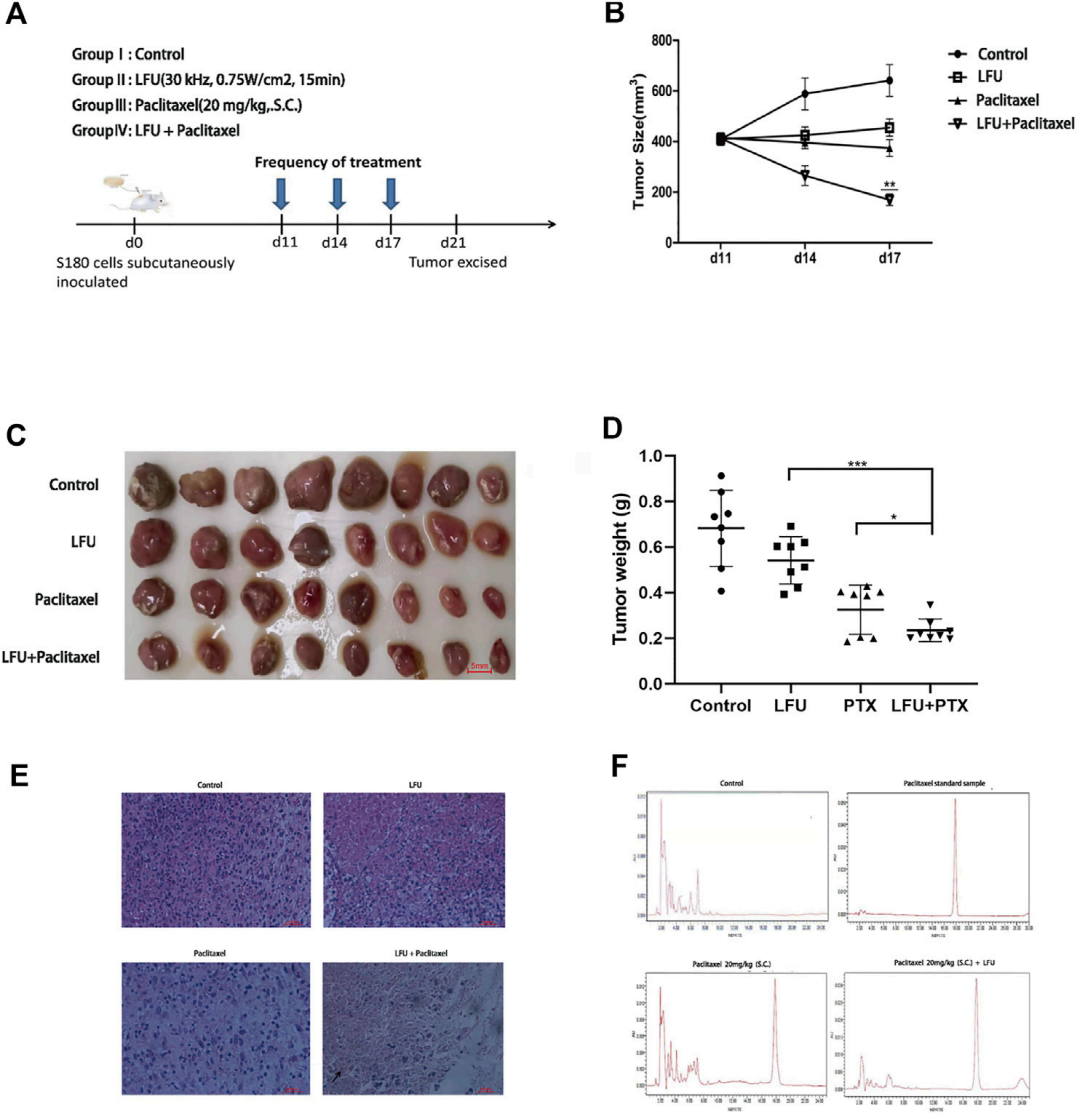
LFU combined with PTX inhibited S180 tumor growth and enhanced the local PTX concentrations of the tumor tissues *in vivo* **(A)** Therapy flow chart. **(B)** Tumor in each group after resection. **(C)** Changes in tumor volume in each group over time ( $\bar{x}$ ± s). **(D)** Tumor weight in each group ( $\bar{x}$ ± s). **(E)** Tumor tissue HE staining after treatment in four groups. Note: Black arrows represent cell necrosis and nuclear breakdown. **(F)** High performance liquid chromatography assay of S180 tumors treated with PTX combined with LFU. *p < 0.05, **p < 0.01, ***p < 0.001, ns not significant.

To investigate the anti-tumor impact of LFU *in vivo*, the xenograft models were created by subcutaneous injection of S180 cells in ICR mice. Figure 5A displayed the time course of frequency of treatment after the initiation of therapy at day 11. By comparing the tumor volume from different groups, we found the tumor sizes in LFU + PTX group were much smaller than other groups (Figure 5B). The size and weight of tumors in LFU + PTX group were markedly reduced than those of PTX, LFU treated alone group (Figures 5C,D). Histopathological observations demonstrated increased cell degeneration and necrosis and high nuclear/cytoplasmic ratio in the tumor tissue of the LFU + PTX group, compared to the PTX, LFU alone and control tumor tissues which showed less degeneration and necrosis (Figure 5E). To study the synergistic effect of LFU on PTX drugs *in vivo*, we isolated tumor mass and analyzed the concentration of drugs in the tissues. The 20 mg/kg PTX in combination with the LFU group accumulated 158.5 mg/g tumor tissue in the S180 sarcoma xenograft, higher than the 70.3 mg/g tumor tissue in the 20 mg/kg PTX group, indicating combination therapy can increase local drug concentration of the treatment tumors. The results suggested that LFU significantly enhanced intracellular concentrations of PTX (Figure 5F).

## Discussion

Sarcomas are lethal tumors that do not respond well to chemotherapy, which remains a widespread and essential treatment for advanced malignant sarcomas (Martin-Broto et al., 2020; Oh et al., 2020). PTX is conventionally used agent for sarcomas, although the effect is often unsatisfactory and requires a higher dose with accompanying adverse effect (Lewcun et al., 2020). Therefore, it is of great significance to enhance the sensitivity of PTX and find effective clinical treatments to improve clinical effectiveness for sarcoma patients. In the current study, we evaluated whether LFU combined with PTX could act as a means of comprehensive treatment for synergistically increasing the anti-tumor action of the drug and providing experimental basis for clinical management of sarcoma tumor. To the best of our knowledge, this study investigated for the first time the potential therapeutic value of LFU in the treatment of sarcoma with PTX. We analyzed the effect of LFU therapy combined with PTX on sarcoma cell by the analysis of the apoptosis and inhibition rate of cell growth *in vitro*, and determined the anti-tumor effect by examining the tumor weight with or without LFU in the sarcoma xenograft model, to demonstrate LFU in combination with PTX in the subcutaneous treatment of sarcoma has important synergistic antitumor effects and further increasing the intracellular concentration of PTX.

Electro-chemotherapy has been used in patients with cutaneous and subcutaneous tumors and has been shown to have antitumor effects, independent of the histology of the tumor (De Angelis et al., 2021). Sonodynamic therapy was introduced in 1989 with the aim of improving the effectiveness of drug therapy and also reducing drug-related systemic toxicity. According to previous studies, ultrasound irradiation can enhance the antitumor effect of chemotherapeutic drugs *in vitro* (Panzone et al., 2022), and the mechanism of increased chemosensitivity of ultrasound-treated cells is not well understood, while cavitation and resealing of cell membranes by acoustic pressure are thought to be the main reasons for increasing the intracytoplasmic concentration of the administered drugs (Carstensen et al., 2000; Maxwell et al., 2013). In this study, CCK8 assays showed that LFU inhibited sarcoma cell growth and proliferation in a dose- and time-dependent manner, and that the combination of LFU with PTX significantly increased the inhibition rate of sarcoma cells. It was shown that the proportion of sarcoma cells in the G2/M phase was essentially unchanged when LFU alone was administered *in vitro*, whereas PTX, when synergized with LFU, blocked the cell cycle of sarcoma cells in the G2/M phase.

In addition, this study examined the effects of LFU and PTX on apoptosis and cell cycle using flow cytometry and Western blot assays. In existing studies, the endogenous mitochondrial apoptotic pathway, the most important apoptotic pathway, has been demonstrated by increasing the disruption of caspase-3 protein or by inhibiting the level of Bcl-2 protein (Wang et al., 2019). In our study, the ratio of Bcl-2/Bax was reduced in sarcoma cells treated *in vitro* with the combination of LFU and PTX, suggesting that the combination of LFU and PTX enhances apoptosis in sarcoma cells. In addition to this, PTX prevents microtubule depolymerization by stabilizing and enhancing microtubule protein polymerization, thus inhibiting mitosis in tumor cells. In our study, the same can be found in sarcoma cells treated with the combination of LFU and PTX *in vitro*, where the cell cycle was substantially blocked in the G2/M phase. We also found that activation of the JAK2/STAT3 signaling pathway, which plays an important role in cell proliferation as a major chain of intracellular signaling, was inhibited during *in vitro* treatment with LFU and PTX, and inhibition of JAK2/STAT3 would result in blocked cell proliferation.

After JAK2/STAT3 inhibition, we observed a decrease in c-MYC protein expression in sarcoma cells treated with LFU and PTX combination *in vitro*. *c-MYC* is one of the common proto-oncogenes and plays an important role as a transcription factor in cell cycle and cell proliferation (Donati and Amati, 2022). Previous studies have shown that MYC can bind directly to the promoter of *PD-L1* gene, while PD-L1 can inhibit T cell responses in the tumor microenvironment, and further found that MYC inactivation in mouse tumors can down-regulate the expression of PD-L1 and enhance anti-tumor immune responses. Therefore, we examined the expression of PD-L1 in sarcoma cells treated with LFU and PTX combination *in vitro* and found that the expression of PD-L1 decreased in the combination group, suggesting that the combination treatment of LFU and PTX may enhance the anti-tumor immune response. Further, we found that the expression of BCL-2 downstream of JAK2/STAT3 was also reduced, and BCL-2 was associated not only with apoptosis but also with the infiltration of immune cells in the immune microenvironment of the tumor (Liu et al., 2022). Therefore, we obtained from the immune infiltration analysis that BCL-2 expression was negatively correlated with the infiltration of immune cells (including T cells, Th1 cells and macrophages, etc.), so the decrease of BCL-2 expression suggested that the infiltration of immune cells might be increased in the combination group, which further inhibited the tumor development.

Finally, our study showed that LFU, especially in combination with PTX, can develop a significant inhibitory effect on the growth of S180 xenografts. *In vivo*, compared with PTX alone, PTX in combination with LFU can markedly reduce the volume of tumor. At the same time, LFU can effectively reduce or inhibit the growth of local sarcoma. This treatment strategy should ideally improve the bioavailability of chemotherapeutic drugs and simultaneously minimize the harmful systemic side effects on the patient (Sorace et al., 2012; Schoellhammer et al., 2015), so it would be anticipated that LFU would enable penetration of drugs deeper into tissues. The use of ultrasound in specific tissues activates drugs to accumulate to higher drug levels in the target cells, thereby reducing the concentration of drugs required to maintain or enhance the effect of the drugs.

LFU is an inexpensive, readily available, relatively safe, non-traumatic, side-effect-free, and non-toxic form of treatment which is also suitable for specific areas deep in the tissue. Therefore, it is expected to be a means to deliver targeted drugs to cancer patients and intensify treatment at specific sites. LFU can significantly decrease the dose of chemotherapy drugs needed to produce efficacy, thereby reducing or eliminating side effects. Ultrasound promotes membrane permeability, which increases intracellular drug accumulation. Now low frequency ultrasound has been used for clinical application in permeable medicine to tumor therapy, tuberculosis therapy, drug resistance of anti-infective and inflammatory bacteria, thrombolytic dialysis agent, osteoarthropathy, soft tissue injury, fracture healing, tumor gene therapy targeting introduction, etc. At present, the key factors that affect the efficacy of chemotherapy are the concentration of drugs used in combination and the adequate exposure time of drugs in specific sites. These findings and data in support of the way of direct local application and warrant in-depth research to determine if this method can inhibit local recurrence and improve clinical outcomes for cancer patients.

## Data Availability

The raw data supporting the conclusion of this article will be made available by the authors, without undue reservation.

## References

[B1] Carstensen E. L. Gracewski S. Dalecki D. (2000). The search for cavitation *in vivo* . Ultrasound Med. Biol. 26 (9), 1377–1385. 10.1016/s0301-5629(00)00271-4 11179611

[B2] Casey S. C. Tong L. Li Y. Do R. Walz S. Fitzgerald K. N. (2016). MYC regulates the antitumor immune response through CD47 and PD-L1. Science 352 (6282), 227–231. 10.1126/science.aac9935 26966191 PMC4940030

[B3] De Angelis B. Balzani A. Pagnotta A. Tati E. Orlandi F. D'Autilio M. (2021). Malignant skin cancer excision in combined therapy with electro-chemotherapy and dermal substitute. Curr. Oncol. 28 (3), 1718–1727. 10.3390/curroncol28030160 34063113 PMC8161833

[B4] Donati G. Amati B. (2022). MYC and therapy resistance in cancer: Risks and opportunities. Mol. Oncol. 16 (21), 3828–3854. 10.1002/1878-0261.13319 36214609 PMC9627787

[B5] Herrmann A. Kortylewski M. Kujawski M. Zhang C. Reckamp K. Armstrong B. (2010). Targeting Stat3 in the myeloid compartment drastically improves the *in vivo* antitumor functions of adoptively transferred T cells. Cancer Res. 70 (19), 7455–7464. 10.1158/0008-5472.CAN-10-0736 20841481 PMC3058618

[B6] Hu Z. Lv G. Li Y. Li E. Li H. Zhou Q. (2016). Enhancement of anti-tumor effects of 5-fluorouracil on hepatocellular carcinoma by low-intensity ultrasound. J. Exp. Clin. Cancer Res. 35, 71. 10.1186/s13046-016-0349-4 27102814 PMC4840943

[B7] Kortylewski M. Kujawski M. Wang T. Wei S. Zhang S. Pilon-Thomas S. (2005). Inhibiting Stat3 signaling in the hematopoietic system elicits multicomponent antitumor immunity. Nat. Med. 11 (12), 1314–1321. 10.1038/nm1325 16288283

[B8] Kortylewski M. Yu H. (2008). Role of Stat3 in suppressing anti-tumor immunity. Curr. Opin. Immunol. 20 (2), 228–233. 10.1016/j.coi.2008.03.010 18479894 PMC2488961

[B9] Lewcun J. A. Pameijer C. Kass R. Cream L. Hershock D. Brooks A. J. (2020). Doxorubicin, paclitaxel, and cisplatin based chemotherapy for the treatment of angiosarcoma: Two case reports. Int. J. Surg. Case Rep. 68, 83–87. 10.1016/j.ijscr.2020.02.036 32120283 PMC7052479

[B10] Liu L. Cheng X. Yang H. Lian S. Jiang Y. Liang J. (2022). BCL-2 expression promotes immunosuppression in chronic lymphocytic leukemia by enhancing regulatory T cell differentiation and cytotoxic T cell exhaustion. Mol. Cancer 21 (1), 59. 10.1186/s12943-022-01516-w 35193595 PMC8862474

[B11] Martin-Broto J. Moura D. S. Van Tine B. A. (2020). Facts and hopes in immunotherapy of soft-tissue sarcomas. Clin. Cancer Res. 26 (22), 5801–5808. 10.1158/1078-0432.CCR-19-3335 32601077 PMC7669707

[B12] Maxwell A. D. Cain C. A. Hall T. L. Fowlkes J. B. Xu Z. (2013). Probability of cavitation for single ultrasound pulses applied to tissues and tissue-mimicking materials. Ultrasound Med. Biol. 39 (3), 449–465. 10.1016/j.ultrasmedbio.2012.09.004 23380152 PMC3570716

[B13] Oh C. R. Hong J. Y. Kim J. H. Lee J. S. Kim H. S. Kim T. W. (2020). Real-world outcomes of pazopanib treatment in Korean patients with advanced soft tissue sarcoma: A multicenter retrospective cohort study. Target. Oncol. 15 (4), 485–493. 10.1007/s11523-020-00731-z 32607656

[B14] Ok C. Y. Chen J. Xu-Monette Z. Y. Tzankov A. Manyam G. C. Li L. (2014). Clinical implications of phosphorylated STAT3 expression in De Novo diffuse large B-cell lymphoma. Clin. Cancer Res. 20 (19), 5113–5123. 10.1158/1078-0432.CCR-14-0683 25124685 PMC4184926

[B15] Panzone J. Byler T. Bratslavsky G. Goldberg H. (2022). Applications of focused ultrasound in the treatment of genitourinary cancers. Cancers (Basel) 14 (6), 1536. 10.3390/cancers14061536 35326687 PMC8945954

[B16] Sadeghi-Naini A. Falou O. Tadayyon H. Al-Mahrouki A. Tran W. Papanicolau N. (2013). Conventional frequency ultrasonic biomarkers of cancer treatment response *in vivo* . Transl. Oncol. 6 (3), 234–243. 10.1593/tlo.12385 23761215 PMC3678128

[B17] Schoellhammer C. M. Schroeder A. Maa R. Lauwers G. Y. Swiston A. Zervas M. (2015). Ultrasound-mediated gastrointestinal drug delivery. Sci. Transl. Med. 7 (310), 310ra168. 10.1126/scitranslmed.aaa5937 PMC4825174 26491078

[B18] Schoellhammer C. M. Traverso G. (2016). Low-frequency ultrasound for drug delivery in the gastrointestinal tract. Expert Opin. Drug Deliv. 13 (8), 1045–1048. 10.1517/17425247.2016.1171841 27049815 PMC5003179

[B19] Shen Z. Y. Shen E. Diao X. H. Bai W. K. Zeng M. X. Luan Y. Y. (2014). Inhibitory effects of subcutaneous tumors in nude mice mediated by low-frequency ultrasound and microbubbles. Oncol. Lett. 7 (5), 1385–1390. 10.3892/ol.2014.1934 24765142 PMC3997662

[B20] Sorace A. G. Warram J. M. Umphrey H. Hoyt K. (2012). Microbubble-mediated ultrasonic techniques for improved chemotherapeutic delivery in cancer. J. Drug Target. 20 (1), 43–54. 10.3109/1061186X.2011.622397 21981609 PMC3417245

[B21] Wang W. Zhu M. Xu Z. Li W. Dong X. Chen Y. (2019). Ropivacaine promotes apoptosis of hepatocellular carcinoma cells through damaging mitochondria and activating caspase-3 activity. Biol. Res. 52 (1), 36. 10.1186/s40659-019-0242-7 31300048 PMC6625015

[B22] Xia C. Y. Liu Y. H. Wang P. Xue Y. X. (2012). Low-frequency ultrasound irradiation increases blood-tumor barrier permeability by transcellular pathway in a rat glioma model. J. Mol. Neurosci. 48 (1), 281–290. 10.1007/s12031-012-9770-0 22528460

[B23] Yang Y. Bai W. Chen Y. Nan S. Lin Y. Ying T. (2016). Low-frequency ultrasound-mediated microvessel disruption combined with docetaxel to treat prostate carcinoma xenografts in nude mice: A novel type of chemoembolization. Oncol. Lett. 12 (2), 1011–1018. 10.3892/ol.2016.4703 27446386 PMC4950530

[B24] Yu H. Lee H. Herrmann A. Buettner R. Jove R. (2014). Revisiting STAT3 signalling in cancer: New and unexpected biological functions. Nat. Rev. Cancer 14 (11), 736–746. 10.1038/nrc3818 25342631

[B25] Yu H. Pardoll D. Jove R. (2009). STATs in cancer inflammation and immunity: A leading role for STAT3. Nat. Rev. Cancer 9 (11), 798–809. 10.1038/nrc2734 19851315 PMC4856025

[B26] Zhang Chen Ouyang L. X. Cheng Liu Cheng Y. Liu B. (2012). Plant natural compounds: Targeting pathways of autophagy as anti-cancer therapeutic agents. Cell. Prolif. 45 (5), 466–476. 10.1111/j.1365-2184.2012.00833.x 22765290 PMC6496896

